# Facial bone fragmentation in blind cavefish arises through two unusual ossification processes

**DOI:** 10.1038/s41598-018-25107-2

**Published:** 2018-05-03

**Authors:** Amanda K. Powers, Shane A. Kaplan, Tyler E. Boggs, Joshua B. Gross

**Affiliations:** Department of Biological Sciences, University of Cincinnati, 45221 Cincinnati, OH USA

## Abstract

The precise mechanisms underlying cranial bone development, evolution and patterning remain incompletely characterised. This poses a challenge to understanding the etiologies of craniofacial malformations evolving in nature. Capitalising on natural variation, “evolutionary model systems” provide unique opportunities to identify underlying causes of aberrant phenotypes as a complement to studies in traditional systems. Mexican blind cavefish are a prime evolutionary model for cranial disorders since they frequently exhibit extreme alterations to the skull and lateral asymmetries. These aberrations occur in stark contrast to the normal cranial architectures of closely related surface-dwelling fish, providing a powerful comparative paradigm for understanding cranial bone formation. Using a longitudinal and *in vivo* analytical approach, we discovered two unusual ossification processes in cavefish that underlie the development of ‘fragmented’ and asymmetric cranial bones. The first mechanism involves the sporadic appearance of independent bony elements that fail to fuse together later in development. The second mechanism involves the “carving” of channels in the mature bone, a novel form of post-ossification remodeling. In the extreme cave environment, these novel mechanisms may have evolved to augment sensory input, and may indirectly result in a trade-off between sensory expansion and cranial bone development.

## Introduction

Despite substantial conservation of skeletal development across vertebrates^1,2^, cranial shape is remarkably flexible^3^. Darwin’s finches^4^ and African cichlids^5^ are classic examples of adaptive changes in cranial shape; both of which have revealed genetic pathways with previously unappreciated roles in craniofacial patterning. The nature of changes to cranial form in the absence of obvious selective pressures, however, remains largely unexplored. This class of morphological aberrations are relevant to humans, given that craniofacial abnormalities are extremely common (1:700 live births), range in severity, have evolved in response to natural environmental pressures, and often require extensive surgical interventions as a course of treatment^6,7^. Understanding how disruptions to developmental processes lead to cranial anomalies is a crucial step in advancing clinical interventions for humans.

**Figure 1:**
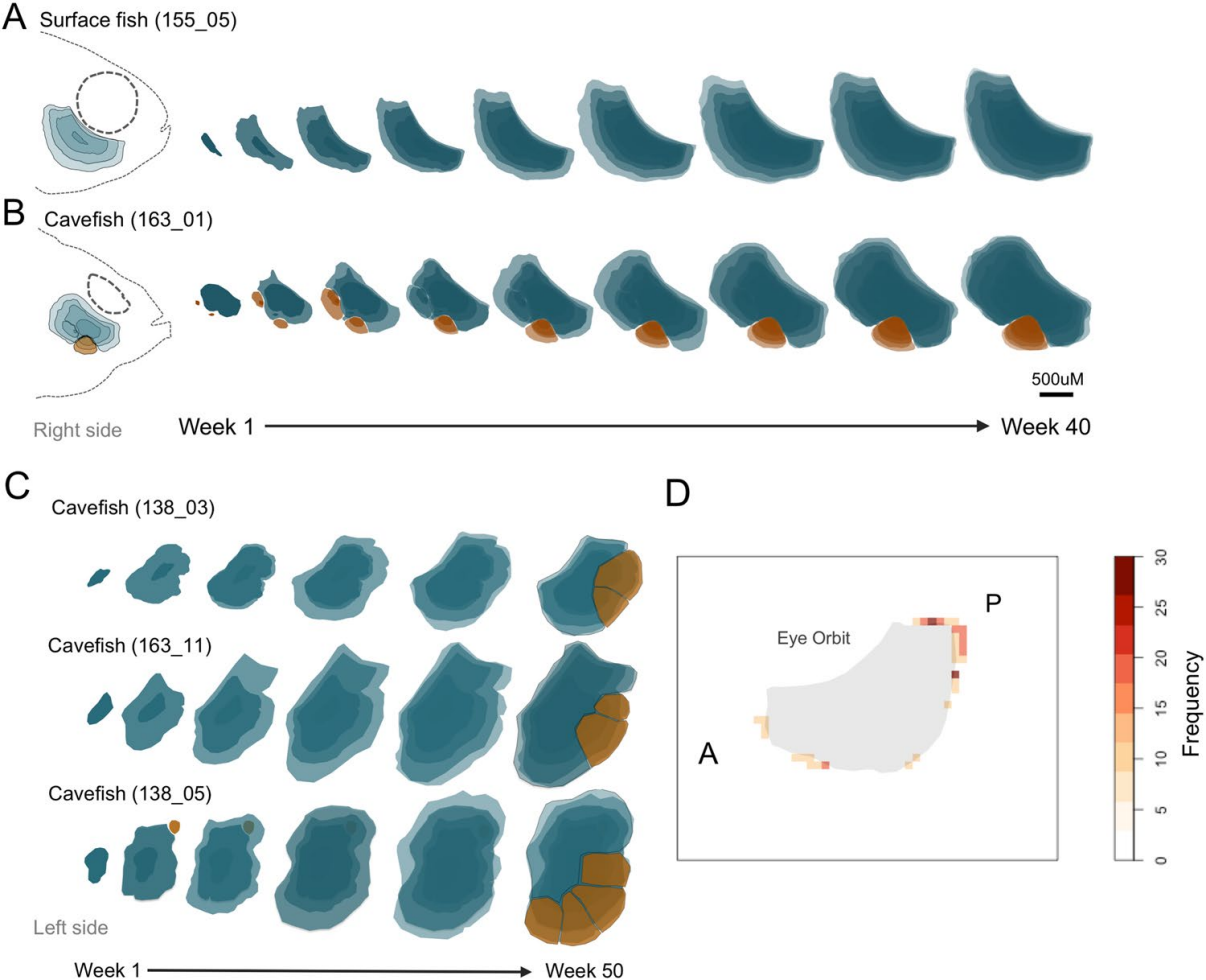
Two distinct developmental mechanisms underlie cranial bone fragmentation in *Astyanax* cavefish. An individual surface fish (155_05) demonstrates normal right-sided SO3 bone growth from a single ossification center (1°OC; blue animation) over 40 weeks (**A**). Cavefish display two novel ossification mechanisms that underlie fragmentation of cranial bones (**B**,**C**). Cavefish specimen 163_01 exhibited a 1°OC (blue) and multiple 2°OCs (orange) on the right side of the face, some of which were resorbed into the 1°OC, while the more anterior 2°OC persisted as a distinct fragment (**B**; mechanism 1). SO3 ossification on the left side of the face for cavefish individuals 138_03 and 163_11 stemmed from a 1°OC, but displayed irregular shape in growth, and eventually underwent post-ossification bone remodeling by 50 wpf, forming fragments (**C**). Cavefish 138_05 displayed both mechanisms, with an early 2°OC in the posterior that was resorbed by 20 wpf and evidence of post-ossification bone remodeling by 50 wpf that resulted in 5 fragments (**C**; mechanism 2). A spatial analysis visualised as a heat map revealed an increase in frequency of bone remodeling initiation sites across cavefish individuals (n = 30) at the posterior margin of the SO3 bone (**D**). The scale bar represents 500 μm.

The blind Mexican cavefish, *Astyanax mexicanus*, harbors numerous cranial anomalies, including several impacting a complex of dermal bones encircling the collapsed eye orbit^8^. These “suborbital” (analogous to infraorbital) bones exhibit fragmentation (Fig. 1B,C) which is marked by the presence of multiple, discrete, smaller bony elements in adults^9,10^. Historical analyses of the holotype specimen^11^ incorrectly attributed bone fragmentation to an injury either sustained in life or during preservation. The discovery of multiple, geographically distinct cave localities in the 1940s revealed that this unusual trait was present across many putatively-isolated populations of cavefish^10,12^. Cranial bone fragmentation most commonly occurs in the third suborbital bone (“SO3”), the largest of the suborbital series^8,13,14^. Alvarez (1947) noted that fragmentation could range in number from 2–10 distinct elements, indicating a wide spectrum of severity in this natural phenotype^10^.

Unlike cavefish, closely-related surface-dwelling fish do not harbor SO3 bone fragmentation (Fig. 1A)^8,12,15^. This enables a powerful comparative approach to understanding the origins of this unusual facial bone morphology. Notably, through juvenile-hood both surface fish and cavefish develop normal and symmetrical cranial cartilages^16^. Therefore, bone fragmentation is associated with mature development of the osteocranium. Mitchell *et al*. (1977) first proposed a mechanism to explain bone fragmentation as an indirect consequence of eye loss and orbital collapse^12^. Accordingly, cave morphs have regressed their eyes over the course of evolution, the orbital region of the skull has collapsed in the absence of a structural eye, and bone fragmentation is merely a consequence of this cranial feature. Testing this idea, Yamamoto *et al*. (2003) explored a potential interaction between eye loss and bone fragmentation using an experimental lentectomy approach^13^. When transplanting a surface fish lens onto a cavefish embryo, they restored the development of an eye in a normally eyeless cavefish. Despite the development of a structural eye and recovery of normal orbital shape, SO3 fragmentation persisted. This suggested that bone fragmentation is *not a simple consequence of eye degeneration*.

In the same study, Yamamoto *et al*. (2003) described the ossification pattern of the suborbital chain in several fixed individuals using Alizarin red dye^13^. The authors discovered that the SO3 develops from a single condensation of mesenchymal cells (i.e., an ossification center, “OC”). Interestingly, cavefish demonstrated multiple secondary or “ectopic” OCs that were never observed in surface fish. The authors suggested these ectopic centers may develop and persist independently, becoming bone fragments in adults. This hypothesis was not comprehensively evaluated, however, owing to the use of specimens fixed at different developmental stages, which limits the ability to connect early ossification events to adult bone morphology.

To understand the complete ossification program governing fragmentation, we implemented live fluorescent imaging across life history in numerous surface and cavefish individuals. Here, we present the first longitudinal study of cranial bone development across ontogeny in cavefish. We discovered that two novel and temporally-distinct developmental mechanisms underlie cranial bone fragmentation in cavefish. A small percentage of bone fragments arise through persistence of ectopic OCs in cavefish, as predicted by Yamamoto *et al*. (2003)^13^. Interestingly, the majority of bone fragmentation occurs much later in juvenile-hood, resulting from dramatic remodeling of ossified bone through focal areas of resorption.

To our knowledge, this is the first description of extensive bone remodeling involved in the formation of dermal cranial bones. This imbalance between bone deposition and resorption in developing cavefish bones employs mechanisms analogous to other hypo-ossification bone disorders in humans, such as osteoporosis. Additional analyses of the genetic regulation of this process will provide insight to the molecular mechanisms underlying these bony abnormalities. This study underscores the importance of longitudinal studies, particularly impacting later life-history events, for understanding complex developmental mechanisms.

## Results

### Two unusual mechanisms underlie facial bone fragmentation in cavefish

#### A rare mechanism for bone fragmentation: Ectopic ossification centers give rise to SO3 bone “fragments”

Dermal bones form directly from neural crest-derived mesenchymal cells^17^. These cells coalesce into skeletogenic condensations, or primary ossification centers (1°OCs), from which nascent bone extends in multiple directions^1,18^. Yamamoto *et al*. (2003) suggested that multiple, ectopic OCs may be responsible for facial bone fragmentation in cavefish^13^. We tested this hypothesis by employing *in-vivo* calcein staining to characterise ossification of the SO3 bone across ontogeny (Supplemental Fig. 1).

Surface fish SO3 bones ossify from a single, 1°OC beginning at approximately 5 weeks post fertilisation (wpf) or 12 mm standard length (SL; Supplemental Fig. 1A). Secondary OCs were *never* observed in developing surface fish SO3 bones (n = 16). In contrast, cavefish SO3 bones are delayed in commencing ossification until approximately 17 mm SL. Further, ectopic or secondary ossification centers (2°OCs) were observed in 43.3% of cavefish (n = 30). These 2°OCs arose spontaneously during a temporal interval ranging from from 5 wpf – 17 wpf. In addition to temporal variation, we found no evidence of a spatial pattern that 2°OCs conform to. Thus, our analysis across cavefish individuals revealed that a stereotypical condition for onset of ectopic OCs does not exist, which is reflected in the variation of fragmentation patterns observed in adults.

Despite the appearance of 2°OCs in nearly half of the juvenile cavefish we analysed, only 13.3% (4/30) adult cavefish *maintained* SO3 fragmentation associated with ectopic OCs into mature bone development. The majority of 2°OCs (66.7%) were resorbed into the larger, continuously growing SO3 element within 4 weeks. However, 33.3% of cavefish with 2°OCs were classified as ‘slow resorbers’ as it took longer than 4 weeks to completely resorb into the larger SO3 element. On average, 2°OCs developed at 17.9 mm SL (10.3 wpf) in cavefish. By 19.5 mm SL (14.5wpf) on average, 2°OCs were resorbed. Generally, 2°OCs that developed earlier during the ossification process (5–9 wpf) were more likely to persist as autonomous fragments into adulthood. Thus, as predicted by Yamamoto *et al*. (2003), secondary sites of ossification participate – relatively infrequently – in the development of facial bone fragmentation^13^.

#### A novel mechanism accounts for the majority of SO3 fragmentation in cavefish: Post-ossification bone remodeling

In each of the 4 cases of cavefish with SO3 fragmentation associated with 2°OCs (13.3%), there were only 2 bones encompassing the SO3 complex: one larger element and one completely separate fragment. Alvarez (1947) observed cavefish SO3 bones with as many as 10 discrete elements^10^. Further, the vast majority of lab-reared, adult cavefish exhibit substantial SO3 fragmentation on, at least, one side of the face. Therefore, we reasoned that additional developmental mechanisms must participate in bone fragmentation in cavefish. Consequently, we extended *in vivo* calcein imaging beyond 15 wpf to discover other potential processes impacting SO3 bone fragmentation in mature cavefish.

Surprisingly, we discovered that around 20 wpf, “channels” began to materialise within cavefish SO3 bones. These channels were eventually ‘carved’ out into distinct, fragmented elements. In many instances, these channels arose at specific sites of irregular morphology (i.e., ‘notches’) observed much earlier in SO3 bone ossification (Fig. 1C). In 27/30 (90%) of cavefish, post-ossification bone remodeling or ‘carving’ resulted in SO3 fragmentation. Between two and five fragments arose in this manner from a single SO3 bone. In contrast, ectopic OCs (mechanism 1) were only ever associated with one bone fragment.

In several cases (9/30), fragmentation patterns arose from both mechanisms (Fig. 1C, Cavefish 138_05) indicating these processes are not mutually exclusive. Only 2/30 instances of post-ossification carving, however, correlated with the positions of 2°OCs that were resorbed. Therefore, the position of resorbed 2°OCs early on does not prefigure locations of SO3 bone remodeling later in development. Interestingly, we found that remodeling, and subsequent fragmentation, is more likely to occur at the posterior margin of the bone (Fig. 1D). Further, several instances (n = 9) of post-ossification bone remodeling were associated with fusions of the SO3 and SO4 bones. Interestingly, these instances were also associated with a more severe fragmentation pattern in adults (p = 0.002). We observed a weak correlation (R^2^ = 0.1959) between bone size and fragment number (i.e. larger bones do not produce more fragments), which implicates other, currently unknown, factors influencing the severity of bone fragmentation in cavefish.

### Cavefish exhibit lateral asymmetry in SO3 bone development

**Figure 2:**
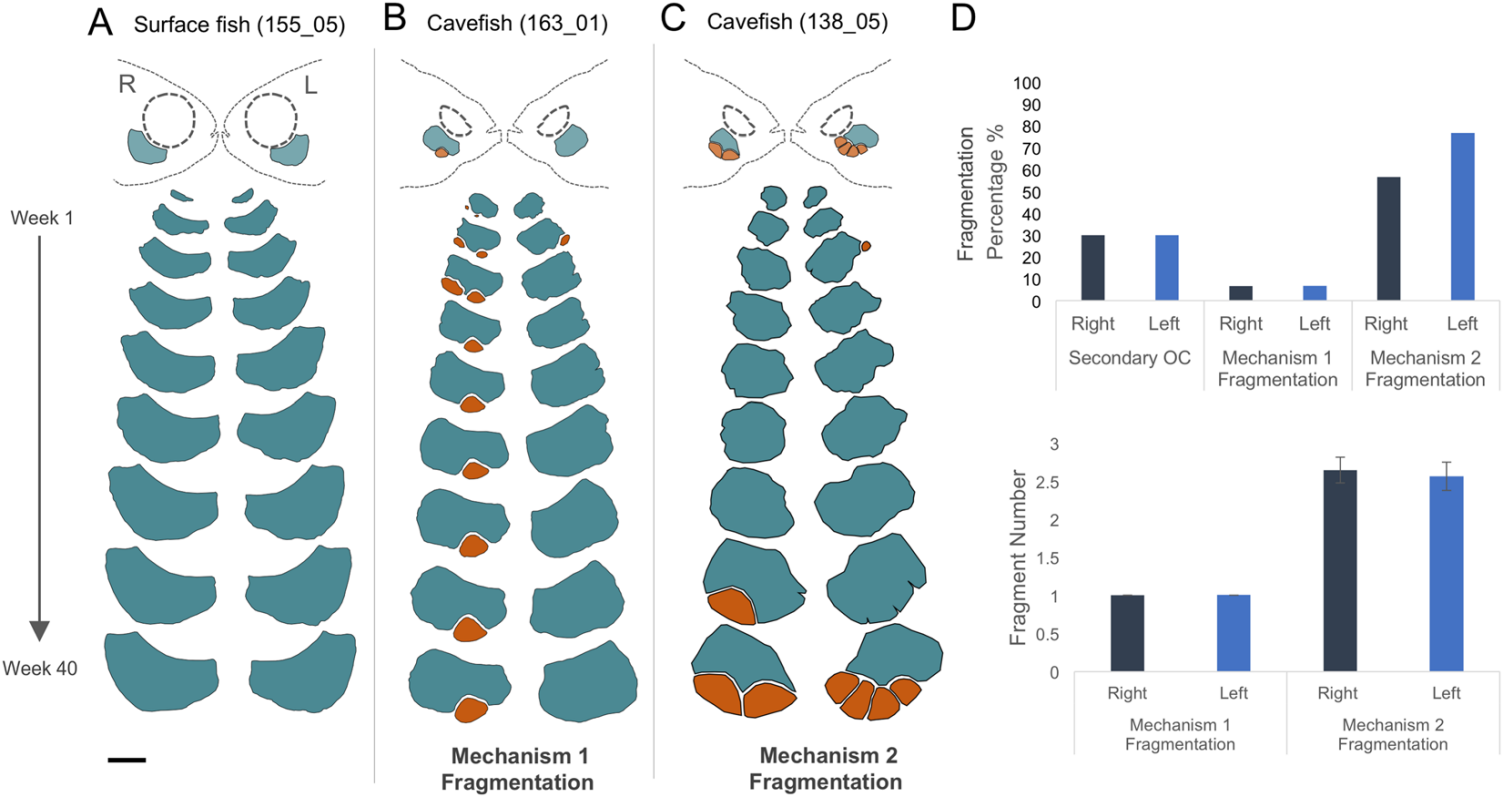
*Astyanax* cavefish exhibit lateral asymmetry in SO3 bone shape and fragmentation during ossification. Lateral ossification of the SO3 bones was observed over a 40-week period. Surface fish (**A**; individual 155_05) undergo symmetrical ossification, both in size and shape of the SO3 bone. Cavefish that display 2°OCs that persist into adulthood as fragments (mechanism 1; individual 163_01) made up 13.3% of the experimental population (n = 30 cavefish) and show lateral asymmetry in the number and positions of 2°OCs, as well as overall SO3 shape throughout ossification (**B**). The majority of the experimental cavefish (90%; n = 30) exhibited fragmentation via post-ossification bone remodeling or mechanism 2 (**C**; individual 138_05). The bone remodeling sites, as well as number and location of fragments, differ between the right and left sides of the face. The frequency of onset of 2°OCs (30%) and mechanism 1 fragmentation (6.67%) were equal on both sides of the face (**D**; n = 30). In mechanism 2, however, the right side (dark blue) of the face showed 56.7% of bone remodeling events while the frequency was higher on the left side (light blue) with 76.7% individuals exhibiting SO3 fragmentation due to post-ossification bone remodeling. There is an increase in the number of fragments for mechanism 2 fragmentation, which produces an average of 2.65 fragments on the right and 2.57 fragments on the left, compared to mechanism 1 fragmentation, which produces just 1 fragment on each side of the face (**D**). The scale bar represents 500 μm.

Cavefish display left-right asymmetry in the number, position, and pattern of 2°OC resorption over the course of SO3 ossification. For example, Individual 163_01 developed a 2°OC on the left side of the face and two on the right, with the most anterior 2°OC persisting as a fragment into adulthood (Fig. 2B). Only 5/30 (16.7%; or 5/13, 38.4% of those fish exhibiting 2°OCs) developed 2°OCs on both sides of the face. The onset of 2°OCs occurred with equal frequency (30%) on each side of the face (Fig. 2D), across the individuals analysed. Similarly, bone fragmentation arising from mechanism 1 (2°OC) occurred with equal frequency (6.67%) on each side of the face (Fig. 2D).

Cavefish also demonstrated asymmetry in post-ossification bone remodeling (mechanism 2), resulting in distinct patterns of fragmentation on each side of the face. Individual 138_05 initially developed a 2°OC on the left side of the face, which was quickly resorbed (Fig. 2C). By 41 wpf (33.1 mm SL), channels began to develop within the SO3 bone on both sides of the face, resulting in 3 fragments on the right side and 5 fragments on the left side of the face. Remodeling occurred at a higher frequency on the left side (76.7%) compared to the right (56.7%) side of the face (Fig. 2D). Further, thirteen individuals (48.1%) demonstrated “carving” on only one side of the face. Two individuals (163_13 and 163_17) demonstrated carving on one side, and a fragment from a 2°OC on the other side of the face. Interestingly, however, there was no difference between the average number of fragments on the right versus the left side across all cavefish individuals by the time the SO3 bone has fully matured (Fig. 2D). Despite similarities in fragment number, the spatial pattern and size of fragments differed between sides of the face and across cavefish individuals. SO3 fragmentation patterns are unique within specimens effectively distinguishing different individuals, similar to human fingerprints.

**Figure 3:**
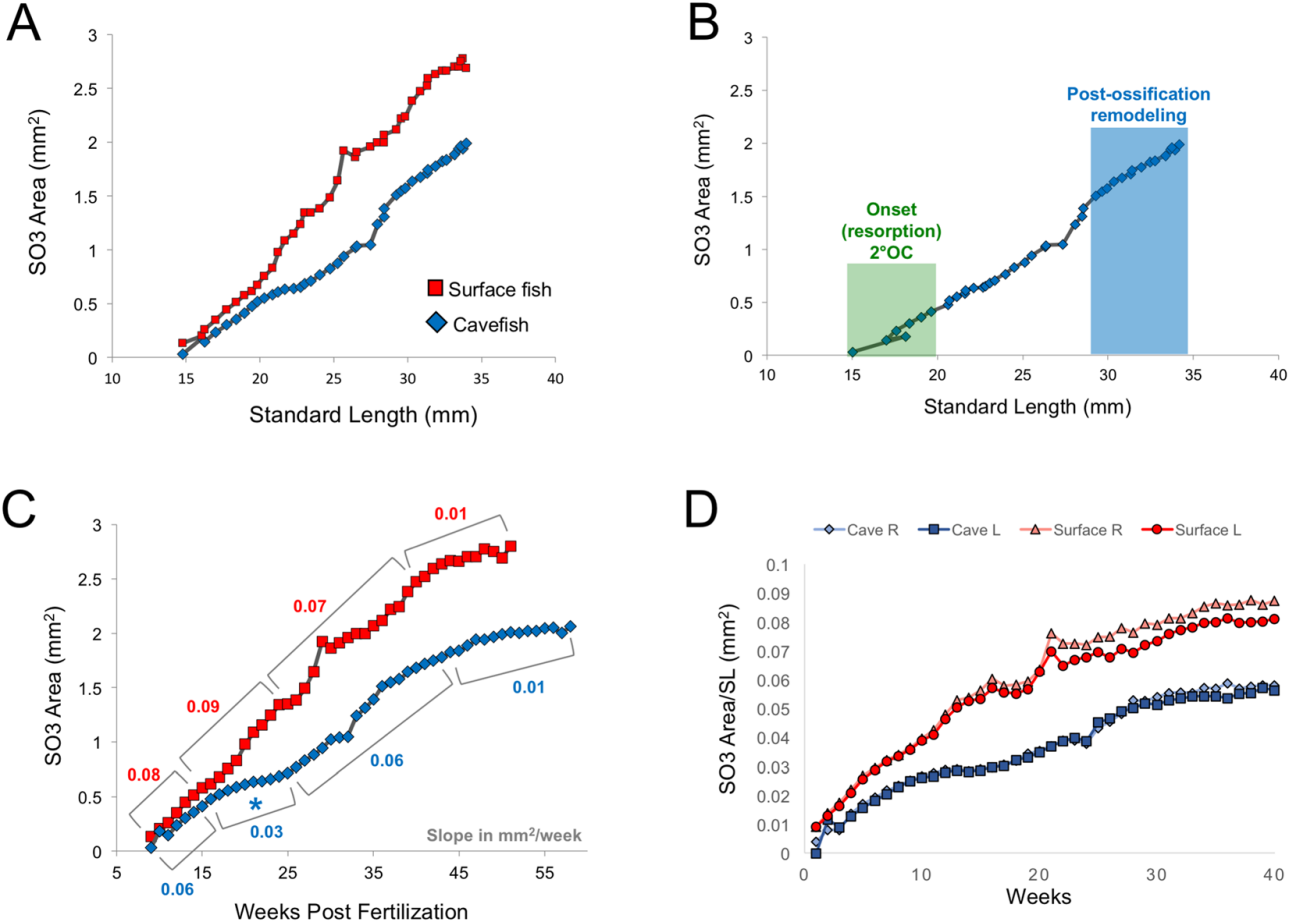
Growth metrics for SO3 ossification in surface and cavefish. There is a linear relationship between SO3 growth (area in mm^2^) and body size (SL in mm) for both surface (red; n = 16) and cavefish (blue; n = 30; **A**). The window for the spontaneous onset and resorption of 2°OCs in cavefish is small, ranging from an average of 10–14.5 wpf or 15–20 mm SL (**B**; green). Ectopic OCs that persisted past this window remained separated fragments when fish reached adulthood. Post-ossification bone remodeling occurred later when the fish reached between 30–35 mm SL or 30–40 wpf (**B**; blue). Surface fish have larger SO3 bones that grow at a faster rate than cavefish across weeks post fertilisation (wpf) (**C**). Cavefish undergo a plateau in SO3 ossification from 15–25 wpf, growing at 0.03 mm^2^/week compared to 0.09 mm^2^/week (**C**). Both morphs reached adult SO3 bone areas at approximately 45 wpf, with diminutive growth thereafter (0.01 mm^2^/week). Average SO3 areas normalised to SL for each morph were measured for both the right and left sides of the face across the number of weeks they were analysed (**D**). There was no appreciable difference between cavefish right (light blue) and left (dark blue) SO3 area over 40 weeks of imaging. There was a slight difference between surface fish right (light red) and left (dark red) areas after 20 weeks of imaging.

Interestingly, when SO3 bony area and associated fragments are accounted for, cavefish demonstrate symmetrical growth on both sides of the face (Fig. 3D). This suggests recruitment of bony progenitors are bilaterally similar, despite dramatic differences in morphology. Surface fish displayed symmetrical patterns of shape and growth of the SO3, until ~20 wpf. Interestingly, after this point in development, we discovered deviations in growth between the right and left sides (Fig. 3D). The relevance or underlying basis for this asymmetric growth is unknown. However, this observation suggests that surface fish demonstrate a normative level of asymmetry in their facial bone deposition and remodeling programs.

### Surface fish SO3 bones grow at a faster rate than cavefish

Both surface fish and cavefish demonstrate isometric growth of the SO3 bone with body size (standard length (SL); Fig. 3A). Surface fish develop larger SO3 bones with an average area of 2.8 mm^2^ at adulthood (1-year post fertilisation) compared to an average of 2.04 mm^2^ (Fig. 3A) in adult cavefish. Based on increased growth in bony area measurements, surface fish bones grew at ~1.5× the rate of cavefish over the course of 55 weeks (Table 1). Surface fish SO3 development maintained a steady growth rate until 45 wpf, when the rate dropped from 0.0654 mm^2^/week to 0.0123 mm^2^/week, indicating mature SO3 development was essentially completed by 45 wpf (Fig. 3C). Similarly, cavefish SO3 growth rate declined from 0.0613 to 0.0115 mm^2^/week after 45 wpf indicating SO3 development ended around the same period of life history in both morphs.

**Table 1:** SO3 bone growth rate (mm^2^/week) in surface and cavefish.

	First 15 weeks	15–25 weeks	25–45 weeks	45–55 weeks	Overall Rate
Surface fish	0.077	0.087	0.0654	0.0123	0.068
Cavefish	0.059	0.027	0.0613	0.0115	0.045
*Rate of higher growth in surface fish bones*	1.3x	**3.2x**	1.1x	1.1x	1.5x

It was noteworthy that cavefish demonstrated a steep decline in SO3 growth between 15–25 wpf, dropping from 0.059 to 0.027 mm^2^/week. This represents a 3.2× slower growth rate compared to surface fish (0.087 mm^2^/week) during the same interval. This corresponds to the end of 2°OC onset and resorption (mechanism 1), and just before post-ossification bone remodeling occurs in cavefish (mechanism 2; Fig. 3B). At present, it is unclear why cavefish SO3 growth declines for this 10-week period, or how this slowed rate of growth is involved in either (or both) bone fragmentation mechanism(s).

### Intramembranous bone remodeling in cavefish is mediated by robust osteoclast activity

Normal bone remodeling balances the activities of bone depositing cells (osteoblasts) and bone resorbing cells (osteoclasts). An imbalance in these processes results in hypo- or hyper-ossification, leading to human clinical disorders such as osteoporosis^19,20^. During bone resorption, osteoclasts attach to bony matrix and secrete tartrate-resistant acid phosphatase (TRAP), which facilitates bone degradation and resorption^21^.

**Figure 4:**
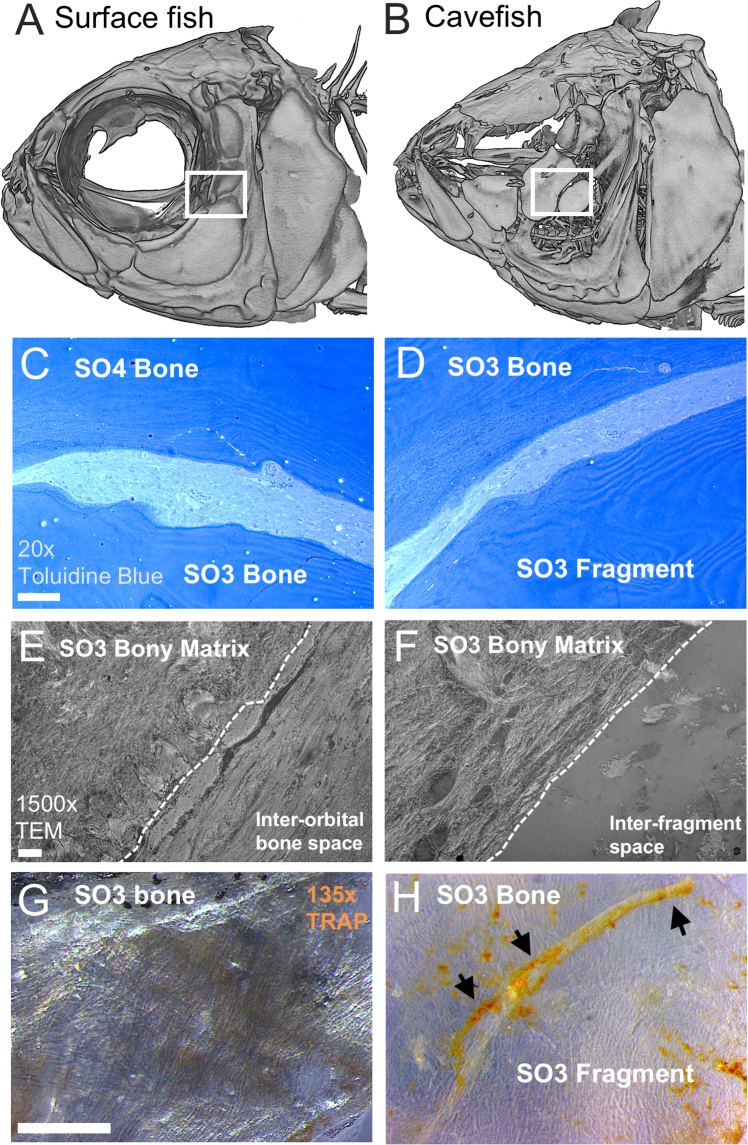
Increased osteoclast activity mediates post-ossification bone remodeling in cavefish SO3 bones. Micro-computed tomography (micro-CT) rendered images illustrate surface (**A**) and cavefish (**B**) skull morphology. White boxes represent dissection of the 1 mm × 1 mm area inclusive of the SO3, inter-orbital space and SO4 bone in surface fish (**A**) and the SO3 and fragmented element in cavefish (**B**). Tissue sections mounted to slides were stained with 1% toluidine blue to illustrate complete separation between the SO3 and SO4 bone in surface fish (**C**) and the SO3 bone and SO3 fragmented element in cavefish (**D**). Transmission electron microscopy (TEM) images reveal histological similarities in SO3 bony matrix between surface (**E**) and cavefish (**F**). Interestingly the inter-orbital space between the SO3 and SO4 bone (**E**) is similar to the inter-fragment space between the SO3 and SO3 fragmented element (**F**) filled with connective tissue and collagen fibers. Staining for osteoclast activity on the SO3 bone with tartrate-resistant acid phosphatase (TRAP; orange) revealed nominal TRAP-positive staining in the surface fish SO3 bone (**G**) and an aggregation of TRAP forming a channel through the cavefish SO3 bone (**H**; black arrows). White scale bars represent 1 mm (**C**–**D**), 2 μm (**E**,**F**), and 250 μm (**G**,**H**).

Whole-mount staining for osteoclasts in dissected SO3 bones revealed substantially elevated TRAP activity in cavefish SO3 bones (Fig. 4H). TRAP-positive staining aggregated in “channels” where bone fragments were actively separating from the larger element, suggesting osteoclasts mediate bone remodeling of SO3 bones in cavefish (i.e., mechanism 2). The lower level of TRAP staining observed in surface fish SO3 bones likely reflects normative levels of bone remodeling (Fig. 4G).

At the ultrastructural level, transmission electron microscopy (TEM) imaging revealed that the larger SO3 element is completely separated from the bony fragment(s), with clusters of collagen fibrils and connective tissue between the elements (Fig. 4F). Interestingly, the inter-fragment area resembles the area between suborbital bones in surface fish (Fig. 4E). This suggests that the process of bone fragmentation leads to the formation of bony fragments that are structurally identical to distinct, mature bones.

## Discussion

Here we present a novel longitudinal study for intra- and inter-individual comparison of cranial bone growth. Facial bone fragmentation in cavefish occurs through two unusual developmental mechanisms: the first is the spontaneous appearance of ectopic, 2°OCs, some of which persist as separate bony fragments into adulthood. While the underlying cause of ectopic bone development in cavefish remains unclear, genetic changes in other systems have resulted in ectopic cranial tissues. For example, mice with null mutations for *Hoxa-2*, *Dlx-2*, *MHox* and *RAR* genes produce ectopic cartilages^22^. Future studies are needed to understand the genetic regulators underlying ectopic OC development.

The majority of 2°OCs fused with the larger SO3 element, while few remained autonomous as bone fragments. Ultrastructural analyses revealed that the inter-fragment region (space between the SO3 bone and fragment) resembled the inter-bone regions between other suborbital bones (Fig. 4C–F). This suggests that certain ectopic OCs may retain cellular signaling programs such that they remain autonomous, similar to other elements in the suborbital chain. The failure of ectopic OCs to fuse to the larger SO3 element in cavefish is an interesting developmental phenomenon and may provide an effective natural model for understanding the cellular origins of human craniofacial abnormalities. This may also clarify differences in cranial development across vertebrates. For instance, the fused frontoparietal bone arises through fusion of bony condensations in chick, while the frontal and parietal bones remain autonomous in mouse^23^.

The second developmental mechanism underlying bone fragmentation is a novel process of post-ossification remodeling. This process resembles developmental features of the patella, an endochondral bone of the lower limb. Early on, the patella is fused to the femur, but later in development is liberated through the combined action of molecular and physical forces^24^. While this is a comparable example, the patella arises as a sesamoid bone embedded in the patellofemoral joint rather than intramembranous ossification (i.e., SO3 bone in cavefish), with a cartilaginous layer residing between the patella and femur. The patella also functions in the mechanical bending of the knee during locomotion^25^, whereas the mechanical forces exerted on the SO3 bone (if any) remain unknown.

It is unclear if SO3 fragmentation is an adaptive trait in cavefish. However, the occurrence of fragmentation across multiple, geographically distinct populations^8,10^ suggests that this trait did not arise as a consequence of genetic drift or neutral mutation. Rather, this trait may have evolved through *indirect selection*^26^ as a trade-off between sensory enhancement and bone ossification. In support of this notion, cavefish exhibit a ~two-fold increase in the number of superficial neuromasts, i.e., mechanosensory receptors that comprise the lateral line system. Yoshizawa *et al*. (2012) found that neuromasts residing on the SO3 bone mediate vibration attraction behavior (VAB) in cavefish^27^. Surface fish do not display this behavior nor do they exhibit SO3 fragmentation, which suggests that fragmented bones may amplify sensory signals as a means of navigating and foraging in the complete darkness of the cave. Gross *et al*. (2016) discovered that superficial neuromasts positioned on the SO3 bone mirror the lateral asymmetry observed in SO3 bone fragmentation^28^. Thus, bone fragmentation occurs in a region of the face that is densely populated with sensory structures, and the asymmetry of these structures mirrors the asymmetry of SO3 bone morphology.

The adaptive relevance of this decoupled facial symmetry is unclear, however cranial asymmetry has been reported in other teleost systems. For instance, lateralisation of the premaxilla in scale-eating cichlids and subsequent feeding behavior has been tied to asymmetries in neuroanatomy^29^. In zebrafish, *fgf8* is a potentially key regulator in laterality since *fgf8*-morphants develop asymmetric pharyngeal arches caused by a break in early molecular signalling^30^.

A potentially adaptive benefit of asymmetric facial bone structure in cavefish comes from prior morphological studies. Powers *et al*. (2017) discovered a dorso-cranial bend in the adult cavefish skulls biased in toward the left^16^. This slight leftward bend in the skull may serve to increase exposure of the *right side of the face* (including the SO3 bone and vibration-sensitive neuromasts) to hydrodynamic water flow^31^. Moreover, the right side of the cavefish skull has been the subject of a number of classical and contemporary analyses in cavefish. For instance, Mitchell *et al*. (1977) first noted that *right-sided* fragmentation could be used to distinguish different cavefish population in a discriminant analysis^12^. Importantly, the left side of the skull was uninformative in the same analysis. Gross *et al*. (2014) discovered a significant genetic association for SO3 bone fragmentation only when scored on the *right side* of face^14^. This may indicate variable penetrance of the trait, or a distinct underlying genetic signal may be associated with the two fragmentation mechanisms described here. Collectively, these phenomena may help explain the ‘genetic asymmetry’ of fragmentation. Future studies will help clarify the presence and potential role of lateral molecular signalling on developmental patterning of the cranium.

The vast majority of cavefish demonstrate bone fragmentation via late bone remodeling mediated by osteoclasts (Fig. 4I). This imbalance between bone deposition and resorption resembles osteoporosis in humans^19,20^, although not perfectly. While osteoclasts mediate osteoporosis, this typically occurs in endochondral bones, wherein the trabecular bone is lost resulting in weakened, porous bones, without separating completely^19^. Further, SO3 fragmentation appears in a highly specific pattern (Fig. 1C), rather than random clusters of bone remodeling. Interestingly, Wada *et al*. (2014) discovered that the trunk canal, an ossified structure in which large canal neuromasts reside, develops from scales through a bone remodeling processes mediated by osteoclasts^32^. This suggests that underlying tissues, e.g., nerve fibers, may recruit osteoclasts as a novel mechanism for developmental patterning through bone resorption. The tissue and/or cellular source of this signal remains a mystery. However, this mechanism occurs in virtually every specimen. Therefore, cavefish can serve as a valuable, natural model for understanding the developmental and genetic mechanisms of bone resorption in the context of aberrant processes impacting other vertebrates, including humans.

## Methods

### Animal Husbandry and Breeding

Animal husbandry and experiments were performed in accordance with the Guide for the Care and Use of Laboratory Animals (NIH) and were approved by the University of Cincinnati Institutional Animal Care and Use Committee (IACUC Protocol # 10-01-21-01). Animals were maintained in a satellite aquatic facility at the University of Cincinnati (Cincinnati, OH, USA). Adult *Astyanax* fish were housed in either 5- or 10-gallon glass tanks on an aquatic husbandry system that circulates reverse-osmosis water with a pH of 7.4 (±0.2) and a conductivity of ~700 μS (±50). System water is circulated through an automatic filtration system including mechanical, biological, micron and UV filters. All fish were subjected to a 12 hr: 12 hr light/dark cycle with a controlled system water temperature (23 ± 2 °C) and fed once daily with a slurry of dry flake food (TetraMin Pro) mixed in system water.

Adult fish were assigned to a rotating breeding schedule adapted from Borowsky^33^. Embryos were reared in a 5-gallon tank off the system with an air bubbler and heater set to 24 °C until they reached ~15 mm SL. Embryos were collected from n = 16 surface fish and n = 30 cavefish originating from the Pachón cave locality (Sierra de El Abra region, Mexico). Pachón cavefish were collected from two pedigrees: ‘Asty-138’ (n = 11) and ‘Asty-163’ (n = 19). Surface fish used in this study (pedigree ‘Asty-155’) were relatives of individuals collected from Río Sabinas and Río Valles drainages near Ciudad Valles, México. Adult fish from these pedigrees were generously provided by Dr. Richard Borowsky (NYU).

At approximately 15 mm SL, juvenile cave and surface fish were housed in individual 1L BPA-free plastic tanks off of the system in order to facilitate staining and maintain identity across our longitudinal study. An important caveat of this rearing method was that *Astyanax* fish demonstrate space-dependent growth^34^, which may have impacted the rate of development. Tank cleaning and system water changes were administered weekly.

### Vital Staining with Calcein for *in-vivo* bone labelling

The purpose of this study was to perform a long-term longitudinal analysis of cranial bone ossification. Chromatic dyes and x-ray or CT imaging have been used to analyse bone in the past^35^. While these methods allow for the visualisation of bone and other structures, they cast limitations on resolution and organisms typically need to be sacrificed. To address these limitations, we co-opted an *in vivo* staining procedure using calcein, a compound that binds to calcified bony matrix and can be visualised under a 488 nm fluorescent filter^35^. Calcein is a more sensitive and inclusive method for staining bone than other chromatic stains (i.e. Alizarin red). We observed no negative effects on growth or development among calcein stained individuals. This conforms to prior studies performed in rainbow trout, immersed in calcein for a period of 6 months, wherein the authors found no evidence of deleterious effects^35^.

Juvenile fish were stained overnight with 0.002 M Calcein (Sigma Aldrich C0875), buffered with NaOH in system water, within their individual 1 L tanks. Following overnight staining, fish were placed in new 1 L tanks with system water to rinse for 1 hour. Fish were anesthetised for imaging by placing them in ice-cold system water for approximately 15 seconds. After lateral images were collected from the right and left sides of the face, fish were measured for SL and revived in system water. Individuals were imaged on a 2% agarose bed at 35× magnification under the GFP (488 nm) fluorescent filter on a stereomicroscrope (Leica, Wetzlar, Germany). Images were collected using varying exposure levels (300–600 ms) owing to minor differences in calcein staining sensitivity across individuals. SO3 bony area (mm^2^; inclusive of bone fragments in cavefish) was measured on both the right and left sides using the ‘Analysis Measurement’ tools in the Leica Application Suite (LAS v3.8). Juvenile fish were imaged once a week from the onset of SO3 ossification (22 mm SL) to mature formation of the SO3 bone (~35 mm SL).

### Chromatic Staining

Whole-mount tartrate-resistant acid phosphatase (TRAP) staining was used to detect osteoclast activity during post-ossification bone remodeling. SO3 bones were dissected from anesthetised fish (1% MS-222) and fixed in 4% paraformaldehyde for 2 hours at room temperature (RT). TRAP staining was performed on whole-mount bones according to Edsall & Franz-Odendaal^21,24^. Following staining, bones were cleared with 1% H_2_O_2_ and imaged using a Leica stereoscope (M205FA).

### Transmission electron microscopy

Facial bone structure of representative surface and cavefish was visualised using high-resolution micro-computed tomography (micro-CT; Fig. 4A,B) imaging according to methods in Powers *et al*. (2017). The complete suborbital bone series was dissected from anesthetised fish (1% MS222) and fixed in 3% glutaraldehyde in cacodylate buffer for 2 hours at RT. Bones were digested in 1% trypsin, cut into 1 mm × 1 mm squares and decalcified in 10% EDTA overnight at RT. Tissue sectioning and imaging were performed at the Cincinnati Children’s Hospital Medical Center (CCHMC) Pathology research core. A subset of sections were stained with 1% Toluidine blue for tissue contrast (Fig. 4C,D), and histological features were characterised according to Rhodin (1974)^36^.

### Statistical analysis

Data analyses were performed using Microsoft Excel for Mac (v15.38) and StatPlus:mac LE (v6.2.21). A spatial heat map of bone remodeling was created using custom R code (Fig. 1D; R Studio v1.0.143)^37^. X and y Coordinates were recorded for each remodeling event observed in reference to the position of the SO3 bone (FIJI v1.0). Coordinates from the right side of SO3 bones were reflected to overlay data for the left side. An empty plot was divided into bins in R. The number of bins was established in order to preserve the 5:3 ratio of pixels in the images. The number of coordinates in each bin were counted and visualised as a heat map using the Spatstat package (v1.51-0). Permutations (10) increased amount of variance among bins enabled better color distinction in the plot, while accurately preserving the proportion of carving events by position.

### Data availability

The authors declare that all data files supporting this study are included in the Supplementary Information files.

## Electronic supplementary material

Supplementary Information

Supplemental Dataset
