# Supplementary material for: Facial bone fragmentation in blind cavefish arises through two unusual ossification processes
Source: Sci Rep. 2018 May 3;8:7015. doi: 10.1038/s41598-018-25107-2 (PMC5934472; doi:10.1038/s41598-018-25107-2)
Supplement: Supplementary file 1 — Supplementary Information [file 41598_2018_25107_MOESM1_ESM.pdf]

Supplementary File

Manuscript SREP-17-55630 Revision I

Title: Facial bone fragmentation in blind cavefish arises through two unusual ossification processes

Authors: Amanda K. Powers1, Shane A. Kaplan1, Tyler E. Boggs1 and Joshua B. Gross1*

Affiliation:1 Department of Biological Sciences, University of Cincinnati, Cincinnati, OH, USA 45221

*Corresponding author email: grossja@ucmail.uc.edu

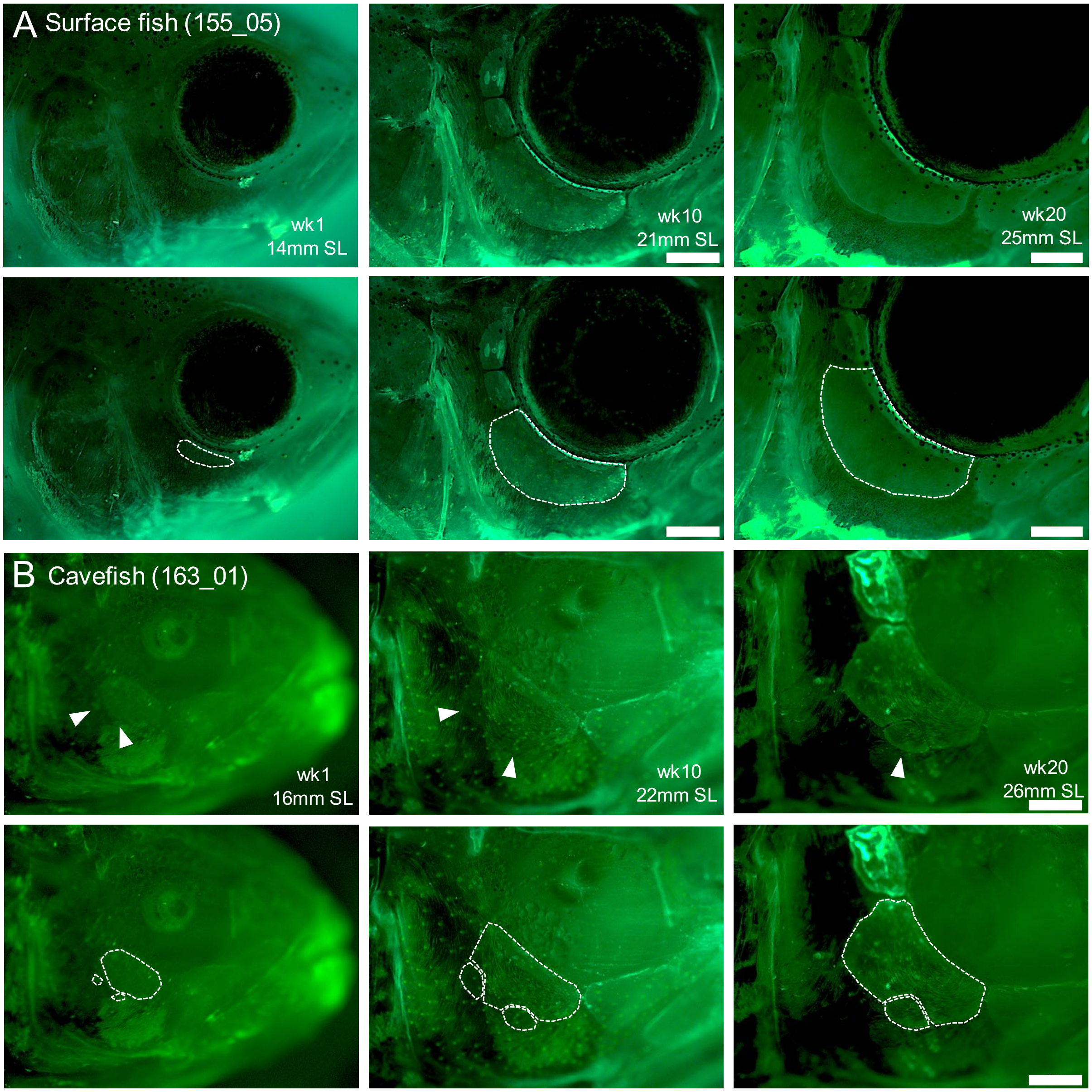

Supplemental Figure 1. Raw images of developing surface fish and cavefish SO3 bones. Surface fish (n=16; individual 155_05 shown in A) stained with calcein were imaged at the onset of SO3 ossification and then each consecutive week for 45 weeks (weeks 1 (14mm SL), 10 (21mm SL) and 20 (25mm SL) represented). The same staining and imaging procedures were used for n=30 cavefish (individual 163_01 shown in in B) over weeks 1 (16mm SL), 10 (22mm SL) and 20 (26mm SL). Secondary ossification centers are denoted with white arrows. Outlines

of the SO3 bones for both surface and cavefish individuals are shown with white dotted lines. Images were captured under the GFP fluorescent light filter at 35x magnification. White scale bars represent 500μm.
